# Chlorpyrifos Exposure and Respiratory Health among Adolescent Agricultural Workers

**DOI:** 10.3390/ijerph111213117

**Published:** 2014-12-16

**Authors:** Catherine L. Callahan, Manal Al-Batanony, Ahmed A. Ismail, Gaafar Abdel-Rasoul, Olfat Hendy, James R. Olson, Diane S. Rohlman, Matthew R. Bonner

**Affiliations:** 1 Department of Epidemiology and Environmental Health, State University of New York at Buffalo, 270 Farber Hall, Buffalo, NY 14214, USA; E-Mail: clc46@buffalo.edu; 2 Community Medicine and Public Health Department, Faculty of Medicine, Menoufia University, Shebin El-Kom 32511, Egypt; E-Mails: manal_1970@yahoo.com (M.A.-B.); aa-ismail@hotmail.com (A.A.I.); gaafar237@yahoo.com (G.A.-R.); 3 Clinical Pathology and Hematology and Immunology, Menoufia University, Shebin El-Kom 32511, Egypt; E-Mail: olfat_hendy@hotmail.com; 4 Department of Pharmacology and Toxicology, State University of New York at Buffalo, 102 Farber Hall, Buffalo, NY 14214, USA; E-Mail: jolson@buffalo.edu; 5 Department of Occupational and Environmental Health, University of Iowa, 145 N. Riverside Drive 100 CPHB Iowa City, IA 52242, USA; E-Mail: diane-rohlman@uiowa.edu; 6 Oregon Institute of Occupational Health Sciences, Oregon Health and Science University, 3181 SW Sam Jackson Park Road, L606 Portland, OR 97239, USA

**Keywords:** chlorpyrifos, lung function, adolescents

## Abstract

Chlorpyrifos (CPF) is a commonly used organophosphate insecticide (OP). In adults, exposure to OPs has been inconsistently associated with reduced lung function. OP exposure and lung function has not been assessed in adolescents. The objective of this study was to assess CPF exposure and lung function among Egyptian adolescents. We conducted a 10-month study of male adolescent pesticide applicators (n = 38) and non-applicators of similar age (n = 24). Urinary 3,5,6-trichloro-2-pyridinol (TPCy), a CPF-specific metabolite, was analyzed in specimens collected throughout the study. Spirometry was performed twice after pesticide application: day 146, when TCPy levels were elevated and day 269, when TCPy levels were near baseline. Applicators had higher levels of TCPy (mean cumulative TCPy day 146 = 33,217.6; standard deviation (SD) = 49,179.3) than non-applicators (mean cumulative TCPy day 146 = 3290.8; SD = 3994.9). Compared with non-applicators, applicators had higher odds of reporting wheeze, odds ratio = 3.41 (95% CI: 0.70; 17.41). Cumulative urinary TCPy was inversely associated with spirometric measurements at day 146, but not at day 269. Although generally non-significant, results were consistent with an inverse association between exposure to CPF and lung function.

## 1. Introduction

Chlorpyrifos (CPF), an organophosphate insecticide (OP), is one of the most widely used insecticides in the United States and worldwide [1]. In 2007, CPF was the most commonly used OP in the United States with an estimated 8 to 11 million pounds applied [2]. The classic mode of action for CPF is to inhibit acetylcholinesterase (AChE), resulting in acetylcholine accumulation in the nervous system [3]. At high doses, the respiratory system is a target for acute OP poisoning [4] via this mode of action. However at levels lower than those known to inhibit AChE and through mechanisms other than AChE inhibition, respiratory effects have been observed in animal studies [5,6,7]. For instance, in a study of guinea pigs, CPF potentiated vagally-induced bronchoconstriction via decreased function of the inhibitory M2 muscarinic receptors on the parasympathetic nerves supplying airway smooth muscle [6]. Animal studies have also suggested that younger animals are less able to detoxify OPs [8,9] and therefore are more susceptible to adverse health effects due to OP exposure. Adolescents may also be susceptible to detrimental effects of pesticides because of their smaller size and higher intake of air per unit of body weight [10,11]. Adolescents’ lungs are not fully developed and may be more vulnerable to insults from inhaled pollutants [12]. For example, previous studies have suggested that exposure to particulate matter is associated with lung function deficits in children and adolescents [12,13]. 

In epidemiologic studies of adults, OP exposure has been associated with self-reported wheeze [3,14,15,16,17] and asthma [18,19]. The relationship between OP exposure and lung function measurements in adults has been equivocal [3,14,20,21,22,23,24,25]. There is a paucity of information about the respiratory effects of pesticides on children [10] and adolescents [26]. To our knowledge, the relationship between CPF exposure and lung function has not been studied in adolescents*.*

Compared with other populations, Egyptian agricultural workers have been observed to have considerably higher levels of exposure to CPF [27]. In Egypt, adolescent agricultural workers apply CPF seasonally, typically during July and ceasing in early August [27]. The objective of this pilot study was to assess the potential association between CPF exposure and reduced lung function. We hypothesized that among adolescents urinary 3,5,6-trichloro-2-pyridinol (TCPy) concentrations would be inversely associated with lung function measurements and pesticide applicators would be more likely to report wheezing than non-applicators.

## 2. Materials and Methods 

### 2.1. Study Population 

A longitudinal study of Egyptian adolescent male pesticide applicators (*n* = 57) and non-applicators (*n* = 38) between the ages of 12 and 21 years was conducted in Menoufia governorate in the Nile Delta north of Cairo. Analyses were restricted to participants 18 years of age or younger to reduce potential confounding by age. Additionally, only participants (*i.e.*, 38 applicators and 24 non-applicators) who completed spirometry were included in these analyses. Applicators are hired seasonally by the Ministry of Agriculture and apply CPF to cotton fields over a period extending from mid-June to early August. Throughout the spray season the Ministry of Agriculture regulates the schedule of application. CPF is the primary pesticide applied. The only other OP insecticide applied is profenofos, which was sprayed for an 8–13 day period after CPF application. Further details regarding the study setting, application process, and biomarker data from this cohort have been described elsewhere [28,29].

Non-applicators were recruited from the same villages as the applicators, but had never worked for the Ministry of Agriculture. Non-applicators were within the same age range as the applicators, although they were not age-matched with the applicators. The study began on 11 April 2010 and ended on 6 January 2011. Over this period, we collected spot urine samples a total of 8 times: (1) day 0 (11 April), (2) day 52, (3) day 73, (4) day 87, (5) day 97, (6) day 111, (7) day 146 (4 September), and (8) day 269 (6 January 2011).

### 2.2. Laboratory Measurements 

Urine samples were collected at field stations for both applicators and non-applicators. Urine samples were placed on wet ice and transported to Menoufia University, where they were stored at −20 °C until they were shipped to the University at Buffalo on dry ice for analysis. Negative-ion gas chromatography-mass spectrometry was used to quantify urinary TCPy, a CPF specific urinary metabolite, as described previously [27]. TCPy values were corrected for creatinine and are expressed as μg TCPy/gm creatinine. Urinary creatinine was quantified using the Jaffe reaction. The within-run imprecision for TCPy analysis was very low as demonstrated by a <2% coefficient of variation and an intraclass correlation coefficient between analytical replicates of 0.997 [30].

### 2.3. Assessment of Lung Function and Covariates

Pulmonary function tests were conducted on day 146, which was after cessation of pesticide applications, but when urinary TCPy levels remained elevated and on day 269 of the study when urinary TCPy levels had returned to near baseline levels. Participants performed up to three maneuvers according the American Thoracic Society guidelines for pulmonary function tests [31] using a Spirolab II spirometer. Percent predicted values were calculated as a percent of the participant measured value to a predicted value of that participant. The predicted values to estimating the predicted value were of Knudson’s method [32]. Percent predicted forced expiratory volume in the first second (FEV_1_) and percent predicted forced vital capacity (FVC) are the primary lung function parameters we report on herein. Restriction was defined as total lung capacity below the 5th percentile and normal FEV_1_/FVC ratio, based on the American Thoracic Society guidelines for restriction [33]. The analyses were restricted to 38 applicators and 24 non-applicators that completed spirometry at the two time points, two participants who were identified as having obstruction were excluded from restriction analyses.

At enrollment, a baseline group-administered interview was conducted to obtain information regarding participants’ age; education; medical history including, physician diagnosed asthma and self-reported wheezing; home and garden pesticide use; exposure to diesel engine exhaust and welding fumes. A baseline health examination was also performed. Additionally, applicators were queried about clothing and personal protective equipment worn during application. The protocol and consent forms used in this study were approved by the Oregon Health & Science University (USA) and Menoufia University (Egypt) Institutional Review Boards. Participants and their legal guardians gave written informed consent prior to enrollment.

### 2.4. Statistical Analysis

Selected characteristics were compared among applicators and non-applicators using Student’s t-tests for continuous variables and chi-square tests for categorical variables. To investigate the association between self-reported wheeze, restriction diagnosed via spirometric measurements, and applicator status, prevalence odds ratios and corresponding 95% confidence limits were calculated via unconditional logistic regression. We considered age, height, weight, mixing pesticides at home, and exposure to diesel engine exhaust as potential confounders. Given our small sample size we were concerned about a sparse data problem that can occur when adjusting for multiple confounders. Our final models were only adjusted for age as the other covariates considered either did not change the point estimate by at least 10% (height and weight) or there were strata with no participants (mixing pesticides at home and diesel engine exhaust).

Cumulative urinary TCPy excretion, over the study period, was estimated by calculating the area under the curve. Each participant’s excretion curve was graphed and integrated using the trapezoid rule to calculate the total TCPy excreted over the course of the study via Stata’s pharmacokinetic function (StataCorp. 2009. Stata Statistical Software: Release 11, StataCorp LP: College Station, TX, USA). Two estimates of cumulative exposure were calculated: (1) up to day 146, which corresponds to the first pulmonary function test and after spraying, but while TCPy levels remained elevated and (2) up to day 269, which corresponds to the end of the study and represents a period when TCPy levels had returned near baseline. Linear regression was used to assess the association between cumulative TCPy excretion and spirometry results while controlling for age, height, weight, pesticide use at home, and exposure to diesel exhaust. To normalize the TCPy variable, a natural log transformation was used. All statistical analyses for this paper were generated using SAS Enterprise Guide (Version 4.3. Copyright © 2006–2010 SAS Institute Inc., Cary, NC, USA), except the integration of the TCPy excretion curves.

## 3. Results and Discussion

### 3.1. Results

Selected characteristics for applicators and non-applicators are depicted in Table 1. Applicators and non-applicators were similar with regards to age, education, and body mass index. Neither the applicators nor the non-applicators reported ever smoking cigarettes or exposure to welding fumes (results not shown). Applicators reported mixing pesticides at home and exposure to diesel engine exhaust more frequently than non-applicators. Applicators had substantially higher levels of urinary TCPy than non-applicators, although non-applicators had detectable levels as well. Applicators tended to have more skin allergies, arthritis, and renal disease than non-applicators, although the small number of these disorders in both groups prohibited further evaluation. Other disorders we considered were diabetes, hypertension, liver disease, heart disease, and epilepsy; these were not present in either group. Most applicators tended to not wear personal protective equipment, although most did report wearing a head covering or shoes at least sometimes. The percent predicted FEV_1_ and FVC were lower among applicators compared with non-applicators, although this difference was not statistically significant. There was only one case of asthma in our sample, precluding statistical analysis with regards to the prevalence asthma and applicator status.

The associations between applicator status and wheeze and restriction are displayed in Table 2. Applicators were more likely to report wheeze and were more likely to be diagnosed with restriction via spirometry. However, these measures were imprecise.

**Table 1:** Selected characteristics of EGAD study participants by applicator status.

		Applicators ( *n* = 38)	Non-Applicators ( *n* = 24)
		Mean (SD)	
Age (years)		15.6 (1.5)	15.4 (2.2)
Education (years)		9.4 (1.9)	9.1 (1.9)
Body Mass Index (kg/m^2^)		20.3 (2.6)	21.2 (3.6)
Cumulative TCPy excretion (μg/gm creatinine)			
Day:			
73		3090.6 (5591.2)	758.0 (537.0)
146		33,217.6 (49,179.3)	3290.8 (3,994.9) *
269		43,063.4 (64,347.8)	5409.9 (6,209.6) *
269–146		1806.8 (2354.2)	3215.2 (4778.2) *
Spirometric measures (% predicted)			
FEV_1_, 1st assessment		98.2 (18.5)	105.4 (17.0)
FEV_1_, 2nd assessment		100.6 (18.3)	105.8 (12.3)
Change in FEV_1_^a^		2.3 (17.3)	−0.7 (19.7)
FVC, 1st assessment		90.1 (22.5)	94.9 (17.6)
FVC, 2nd assessment		88.8 (17.6)	95.2 (12.7)
Change in FVC ^b^		−1.9 (21.5)	−1.6 (21.1)
		Number (%)	
Mixed pesticides at home (yes)		27 (73.0)	10 (47.6) *	
Diesel engine exhaust (yes)		13 (35.1)	3 (15.0)	
Skin allergy (yes)		5 (13.2)	1 (4.2)	
Renal disease (yes)		3 (7.9)	0 (0.0)	
Arthritis (yes)		2 (5.3)	0 (0.0)	
Wears while applying pesticides ^c^			
Respirators		1 (2.6)		
Mask over mouth		4 (10.5)		
Mask over mouth and nose		7 (18.4)		
Waterproof gloves		6 (15.8)		
Googles		4 (10.5)		
Glasses		5 (13.2)		
Shoes or boots (not open sandals)		27 (71.1)		
Head cover/cap		25 (65.8)		

Notes: * *p* < 0.05; Abbreviations: SD, standard deviation; FEV_1_, forced expiratory volume in one second; FVC, forced vital capacity; ^a^ Change in FEV_1_ was calculated by subtracting first assessment of FEV_1_ from second assessment of FEV_1_; ^b^ Change in FVC was calculated by subtracting first assessment of FVC from second assessment of FVC; ^c^ Applicators who reported wearing the item "sometimes" or more were considered an affirmative response.

**Table 2:** Odds ratios and 95% confidence intervals, pesticide applicator status and lung function symptoms.

	Wheeze	No Wheeze	Unadjusted OR (95% CI)	Age-AdjustedOR (95% CI)
Non-applicators	2	22	1.00 (referent)	1.00 (referent)
Applicators	9	29	3.41 (0.67; 17.41)	3.36 (0.65; 17.47)
	Restriction ^a^	Normal		
Day 146				
Non-applicators	7	13	1.00 (referent)	1.00 (referent)
Applicators	17	19	1.66 (0.54; 5.13)	1.71 (0.55; 5.36)
Day 269				
Non-applicators	5	17	1.00 (referent)	1.00 (referent)
Applicators	17	17	3.40 (1.02; 11.32)	3.27 (0.97; 11.08)

Notes: Abbreviations: OR, odds ratio; CI, confidence interval; ^a^ Refers to spirometer identified restriction based on American Thoracic Society guidelines: total lung capacity below the 5th percentile and normal FEV_1_/FVC ratio [33].

**Figure 1:**
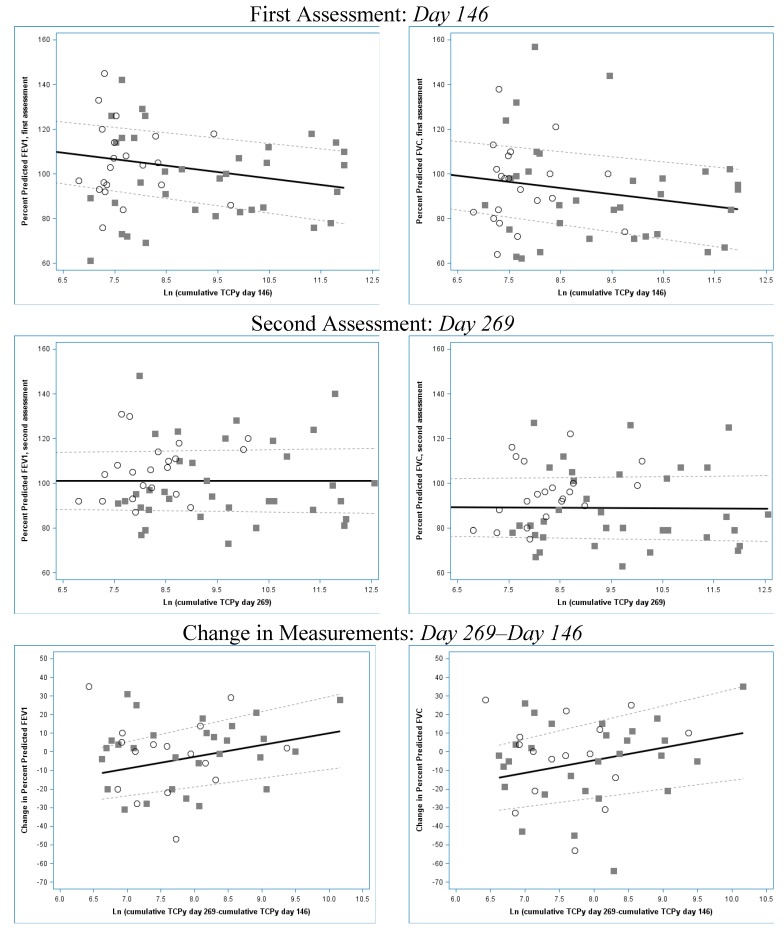
Scatterplots of cumulative urinary 3,5,6-trichloro-2-pyridinol (TCPy) and percent predicted forced expiratory volume (FEV_1_) in one second and percent predicted forced vital capacity (FVC). Models are adjusted for age weight, height, mixing pesticides at home, and diesel exhaust. Applicators are represented by grey squares; non-applicators are represented by white circles; upper and lower confidence intervals are represented by grey dashed lines.

The association between urinary TCPy and spirometric measurements are displayed in Figure 1. Among all participants FEV_1_ and FVC at the first assessment were inversely associated with cumulative urinary TCPy on day 146, which is when TCPy levels remained elevated. On day 269, when TCPy levels had returned to the baseline, cumulative TCPy was not associated with FVC and there was a suggestion of a positive association between cumulative TCPy and FEV_1_, which resulted after multi-variate adjustment. Change in cumulative urinary TCPy from day 146 to day 269 was positively associated with FVC and FEV_1_ among all participants.

### 3.2. Discussion

Adolescent pesticide applicators, who primarily apply CPF, tended to have lower lung function parameters when compared with non-applicators. Additionally, urinary TCPy was inversely associated with spirometric measurements among both applicators and non-applicators, near the end of spraying, but not after TCPy levels returned to baseline, which is consistent with an acute effect of CPF exposure on lung function. However, we are unable to rule out chance as an alternative explanation for our findings, because of the small sample size.

While several studies have examined the effects of organophosphates on wheeze [14,15,16] and lung function in adults [3,14,20,21,22,23,25], there is a paucity of information on the respiratory effects of pesticides in children [10] and adolescents [26]. In our study, adolescent pesticide applicators had 3.4-fold higher odds of reporting wheeze in the past year when compared with non-applicators; this finding is similar in magnitude to a previous study of OP exposure and wheeze in adult non-smoking Costa Rican women [14]. OP exposure has also been reported to be associated with wheeze among adult pesticide applicators exposed to OPs in the Agricultural Health Study (OR applying CPF more than 40 days per year compared with never use = 2.40, 95% CI: 1.24; 4.65) [16,17]. Agricultural workers exposed to OPs in Kenya had higher prevalence of respiratory symptoms than those not exposed. This study defined exposure to OPs as >30% AChE inhibition (prevalence ratio for respiratory symptoms = 2.92, 95% CI 1.12; 7.61) [15]. Among children under age 18 in the United States pesticide use in the kitchen or dining rooms was associated with an increased odds of wheezing (OR= 1.39, 95% CI: 1.08; 1.78) [10] and study of farm workers in Ethiopia age 15–24 found that pesticide applicators had lower spirometric measurements than those who were engaged in farm work but did not apply pesticides [26].

To our knowledge the association between exposures to specific OPs, including CPF, and lung function has not been studied in adolescents. Previous studies of spirometry and OP exposure in adults have been inconsistent. A cross-sectional study of Palestinian farmers found no association between self-reported exposure to dust or pesticides and spirometric measurements [22]. Among 89 greenhouse workers and 25 non-spraying controls in Spain spirometric measurements and exposure to OPs were not associated; OP exposure was defined as a depression of more than 25% in plasma cholinesterase or 15% depression in AChE levels [21]. Self-reported OP exposure was not associated with spirometric measurements in a study of Costa Rican female agricultural workers [14]. Lung function and OP exposure was associated in 25 occupationally exposed Sri Lankan farmers and 22 fishermen who lived within a 25 km radius of fields where OPs were sprayed. The mean FVC for farmers during pesticide spraying was 71.09, 79.79 for the environmentally exposed fishermen and 87.02 for the control group of non-exposed fishermen [20]. A study of Indian agricultural workers found greater than 50% AChE inhibition to be associated with increased reporting of respiratory symptoms and reduced lung function agricultural workers had 13.6% lower mean FVC, and 15.6% lower mean FEV_1_, than non-agricultural workers [3]. Indian OP applicators had a mean peak expiratory flow rate of 395 while non-exposed controls had a mean peak expiratory flow rate of 455 [3].

In our study, FEV_1_ and FVC were inversely associated with cumulative urinary TCPy at the end of spray season (day 146). Although we do not have baseline assessments of lung function, which is a limitation of our study, lung function measurements on day 269 may be an approximation of baseline parameters as urinary TCPy had returned to baseline at this time point. Our observation of an inverse association between cumulative urinary TCPy and spirometric measurements on day 146 coupled with our observation of no association or a slight positive association on day 269 may suggest that CPF exposure has an acute reversible effect on lung function in adolescents. Additionally, we observed a positive association between change in cumulative TCPy and change in lung function measurements, which suggests that those with the highest exposure to CPF during spraying had the greatest increase in lung function parameters when urinary TCPy levels returned to baseline.

Our study has some limitations. Primarily our small sample size limited our statistical power; ability to assess potential effect measure modification, particularly by age; and reduced the number of potential confounders we were able to adjust for. The covariates we did adjust for did not substantially change our measures of association; nevertheless there is still potential for residual confounding. In addition to CPF, applicators may have other workplace exposures that decrease their lung function, such as allergens. There may also be inert ingredients in the mixture applicators used that could adversely affect lung function. However, an inverse association between total urinary TCPy on day 146 and spirometric measurements was present among non-applicators as well suggesting that urinary TCPy is inversely associated with lung function independent of applicator status. Additionally, since this was a repeat-measures study and we observed no association between cumulative urinary TCPy excretion and lung function parameters on day 269, it is unlikely that our observations are confounded by characteristics that presumably remained fixed over the course of study, such as age, height, weight, or household exposures.

Another source of potential bias in this study is a healthy worker effect. Pesticide application is a physically strenuous task with exposure to heat and dust. One might expect that those with reduced lung function would avoid such employment, and those who experienced adverse respiratory effects would terminate employment early. However, we observed that those employed as an applicator had lower lung function parameters than non-applicators, thus bias due to a healthy worker effect is not a likely alternative explanation for our findings.

Spirometry requires some effort on the part of the participant and trained personnel, thus there is some degree of measurement error. Self-reported wheeze is a subjective measure of lung function that could have some degree of measurement error as well [34]. Also, participants reported wheeze on the baseline questionnaire, meaning wheeze could have been present before OP exposure. We expect these errors to be non-differential with regards to exposure status and thus obscuring the association, which may be the most likely explanation for the lack of statistical significance and shallow slopes of regression analyses. Self-reported wheeze and spirometric measurements were not associated in our dataset (results not shown). A previous study of participants who were 18 years old found that remittent wheeze was not associated with spirometric measurements suggesting that wheeze inhibits lung function only during an episode [35]. Therefore we are using two separate measures of lung function, both of which are imperfect, but the errors in measurement are likely not correlated.

Strengths of our study include the use TCPy, a CPF specific metabolite that is often used as a biomarker [36,37], to estimate CPF exposure; excellent quality control in TCPy analysis; the use of spirometry, an objective albeit problematic measure of lung function; a large amount of variability in CPF exposures; and a longitudinal study design with repeat measurements, which allows greater accuracy in estimating CPF exposure and controls for potential confounders that remain fixed during the study period. There was only one case of asthma in our sample, all of our participants were male, and none of our participants reported ever smoking, thus our estimates are likely not confounded by asthma, sex, or smoking. Additionally, our analyses were restricted to participants under age 18 in order to control for potential confounding by age.

## 4. Conclusions

Although our study was small and underpowered for formal statistical testing and lacked a true baseline assessment of lung function, our results are internally consistent that adolescent pesticide applicators have poorer lung function than non-applicators of similar age when lung function is assessed via spirometry and self-report of wheeze. To our knowledge, this is the first study to report on CPF exposure and lung function among adolescents and our results necessarily will require independent replication in a larger study of adolescent pesticide applicators.
